# Initial Real-World Experiences of Pulmonary Vein Isolation and Ablation of Non-Pulmonary Vein Sites Using a Novel Circular Array Pulsed Field Ablation Catheter

**DOI:** 10.3390/jcm13226961

**Published:** 2024-11-19

**Authors:** Joerg Yogarajah, Julie Hutter, Patrick Kahle, Philipp Beaujean, Marko Tomic, Andreas Hain, Samuel Sossalla, Malte Kuniss, Thomas Neumann

**Affiliations:** 1 Department of Cardiology, Kerckhoff Heart and Thorax Center, Campus Kerckhoff, Justus Liebig University Giessen, 61231 Bad Nauheim, Germany; 2 Department of Cardiology, Medical Clinic I, Justus Liebig University Giessen, 35392 Giessen, Germany

**Keywords:** atrial fibrillation, pulsed field ablation, pulmonary vein isolation, left atrial roof ablation, cavo-tricuspid isthmus ablation

## Abstract

**Background and Aims:** Various pulsed field ablation (PFA) systems are currently being developed. Recently, a novel CE-approved circular array PFA catheter (PulseSelect™ PFA System, Medtronic, Minneapolis, MN, USA) was introduced. Data on this commercially available system are sparse. The aim was to elucidate real-world data assessing the feasibility, safety, and acute efficacy of pulmonary vein isolation (PVI) and ablation beyond PVI with this novel ablation system. **Methods:** Consecutive patients with paroxysmal and persistent atrial fibrillation (AF) undergoing first-time ablation with the circular PFA catheter were enrolled in this study. In patients with persistent AF and left atrial (LA) enlargement (LA area > 20 cm^2^), additional left atrial roof ablation (LARA) was performed. Those with concomitant typical atrial flutter received adjunctive cavo-tricuspid isthmus (CTI) ablation. **Results:** A total of 100 AF patients were included (29% female, 50% persistent AF). Of these, 33 patients (33%) underwent adjunctive LARA, 1 patient (1%) received posterior wall isolation, and 6 patients (6%) required additional CTI ablation. The skin-to-skin procedural time averaged 66.3 ± 13.8 min, while the fluoroscopy time and dose area product were 13.7 ± 4.7 min and 6.8 ± 4.9 Gycm^2^, respectively. Acute PVI was achieved in 100% of pulmonary veins. A bidirectional conduction block of the LARA and CTI lines was confirmed in all patients, and no major adverse events were reported. **Conclusions:** These real-world data demonstrate the feasibility, safety, and acute efficacy of PVI and ablation beyond PVI using a novel circular array PFA catheter in patients with atrial fibrillation and flutter. The system can easily be integrated with standard PVI workflows. Further and larger studies are warranted to assess the clinical long-term effectiveness and safety of this PFA system.

## 1. Introduction

Pulsed field ablation (PFA) is a novel non-thermal energy source that has emerged as a promising method for atrial fibrillation (AF) ablation. Unlike traditional ablation techniques like radiofrequency or cryoablation, PFA selectively targets myocardial tissue while minimizing collateral damage to surrounding structures, such as the esophagus or phrenic nerve [1]. This approach could potentially reduce complications associated with thermal ablation. Several PFA systems are currently under development, including the recently introduced CE-approved PulseSelect™ PFA System by Medtronic, which features a circular array catheter [2]. Numerous data exist about the most established Farapulse™ PFA system (Boston Scientific, Marlborough, MA, USA). Pulmonary vein isolation (PVI) and ablation beyond pulmonary veins (PVs), such as posterior wall isolation (PWI), seem to be feasible and safe with this device [3]. The Pulsed AF Pivotal demonstrated similar safety and effectiveness of the PulseSelect™ PFA system compared to established PFA ablation technologies [4,5]. However, data on the real-world application of this system remain limited, especially on ablation beyond PVI. The objective of this study was to provide real-world data on the feasibility, safety, and acute efficacy of PVI and ablation of non-PV-sites using this novel ablation system.

## 2. Methods

### 2.1. Study Population

In this observational, single-center study, 100 consecutive patients were enrolled who underwent first-time AF catheter ablation performed with a novel circular pulsed field ablation device from April to October 2024. Patients with persistent AF and left atrial (LA) enlargement (LA area > 20 cm^2^) underwent adjunctive left atrial roof ablation (LARA). Patients with additional typical atrial flutter were treated with additional cavo-tricuspid isthmus (CTI) ablation. Patients were selected based on consecutive sampling, enrolling each eligible patient during the specified timeframe. Due to the real-world, observational nature of this study, randomization and blinding were not implemented. Instead, our goal was to assess clinical outcomes in a standard clinical practice setting. Exclusion criteria were intracavitary thrombus, prior left atrial ablation, severe valvular heart disease, and pregnancy. This study was approved by the ethics committee of Justus Liebig University of Giessen and registered under No. AZ 83/24. It complied with the Declaration of Helsinki. All patients were informed about the procedure, including ablation beyond PVI, and gave written consent prior to the procedure.

### 2.2. Preprocedural Management

Intracardiac thrombus was ruled out by transesophageal echocardiography in all patients before ablation. Transthoracic echocardiography was performed before ablation to assess the LA area and left ventricular ejection fraction (LVEF). In patients on new oral anticoagulants (NOACs), the drug was discontinued 12–24 h prior to the procedure. In patients treated with vitamin K antagonists, anticoagulation was continued, targeting an international normalized ratio (INR) of 2–3.

### 2.3. Pulsed Field Ablation Procedure

All PFA ablation procedures were performed under deep sedation or general anesthesia, conducted by four different primary operators. A 10-pole diagnostic catheter was placed in the coronary sinus (CS). Following a single transseptal puncture using the Brockenbrough technique (BRK-1) and a transseptal sheath (SL-1), heparin was administered to maintain an activated clotting time (ACT) of over 300 ms. An exchange wire was positioned in the left superior pulmonary vein (LSPV), and the transseptal sheath was exchanged for a 10 Fr steerable sheath (FlexCath Contour™, Medtronic, Minneapolis, MN, USA).

Pulmonary vein angiography was performed to visualize the LA and PV anatomies. Using the steerable sheath, the over-the-wire PFA catheter (PulseSelect™, Medtronic, Minneapolis, MN, USA) was advanced into the left atrium and positioned within the PVs to record electrical PV signals. The position was assessed by fluoroscopy. The ablation catheter features a 9 Fr shaft with bidirectional steering and a 25 mm diameter loop equipped with 9 electrodes, capable of sensing, pacing, and ablating. In total, at least four antral and four ostial applications were delivered. Each application consists of four biphasic, bipolar pulse trains. After each application, the PFA catheter was circumferentially rotated in four different positions to achieve complete PVI, addressing potential gaps between electrodes 1 and 9. The entrance and exit blocks of the PVs were then confirmed using the PFA catheter in sinus rhythm. Before and after administering the PFA applications to right PVs, a low-voltage electrical pulse was delivered from all nine electrodes to test for phrenic nerve capture.

**Figure 1:**
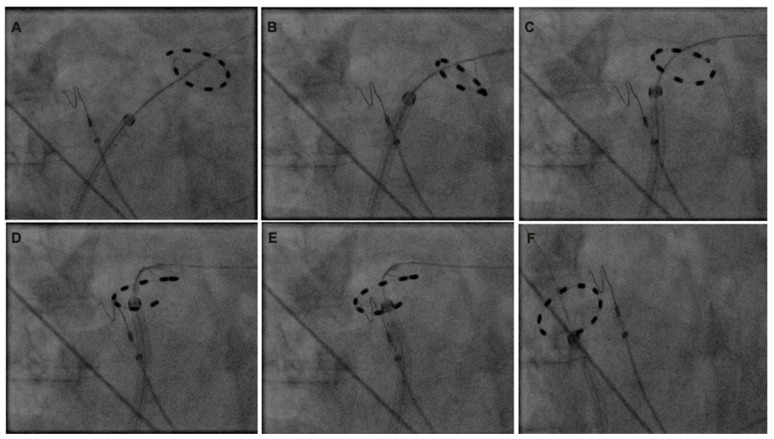
Maneuvers for left atrial roof ablation with circular pulsed field ablation catheter. (**A**): Position during isolation left superior pulmonary vein. (**B**–**E**): Incremental advancement by slight clockwise rotation and slight sheath retraction for generation of roof line. (**F**): Position during isolation of right superior pulmonary vein.

In patients who underwent additional LARA following PVI, the PulseSelect™ catheter was deeply positioned in the left superior pulmonary vein (LSPV) (Figure 1). Sequential overlapping applications were performed along the LA roof, starting near the LSPV isolation site. The PFA catheter was incrementally repositioned by retracting the sheath and slightly rotating the PFA catheter until the isolation position for the right superior pulmonary vein (RSPV) was achieved. The procedural endpoint was defined as complete PVI and conduction block across the LA roof, tested in the sinus rhythm. For patients who continued to experience AF after the initial ablation, electrical cardioversion was performed. Following successful conversion to the sinus rhythm (SR), PVI and LA conduction block were confirmed. The bidirectional block of the LA roof was verified by differential pacing. The PFA catheter was positioned at both the caudal and cranial aspects of the posterior LA wall. Baseline pacing during the SR was conducted at the right atrial upper septum with a cycle length of 500 ms. Activation times at the caudal and cranial positions of the posterior LA wall adjacent to the LA roof were measured. The presence of the LA roof conduction block was confirmed by observing caudocranial ascending activation at the posterior LA wall and a conduction delay of greater than 150 ms next to the ablation area. In patients who underwent PWI, the superior portion of the ablation was performed similarly to the LARA technique. For the inferior part of the LA PWI, ablation began near the left inferior pulmonary vein (LIPV) with overlapping applications, progressing toward the position of the right inferior pulmonary vein (RIPV).

**Figure 2:**
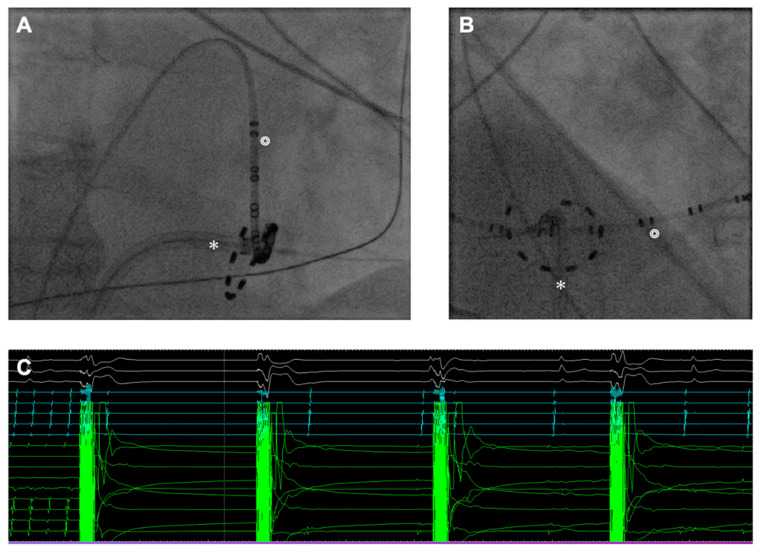
Cavo-tricuspid ablation with circular pulsed field ablation (PFA) catheter. (**A**): Position during ablation of cavo-tricuspid isthmus in right anterior oblique (RAO) view. (**B**): Position during ablation of cavo-tricuspid isthmus in left anterior oblique (LAO) view. (**C**): A case of real-time termination of typical counterclockwise atrial flutter. The atrial flutter terminated after the second PFA application. Green signals are potentials recorded by electrodes of the PFA catheter (✻). Blue signals are recorded by the 20-pol Halo catheter (◎).

In cases where additional atrial flutter was present, a 20-pole diagnostic catheter was placed alongside the tricuspid valve annulus in the CS. Before CTI ablation in each patient, 0.1 mg intravenous nitroglycerin was administered to prevent a coronary artery spasm. CTI ablation was performed by overlapping PFA deliveries to create a CTI line of the block (Figure 2). After each energy delivery, a 12-lead ECG was compared with the baseline ECG to detect conduction disturbances and ST elevations. Afterwards, a bidirectional block was evaluated by pacing both lateral and medial to the CTI line of the block.

### 2.4. Postprocedural Management

Immediately after ablation, transthoracic echocardiography was performed to rule out pericardial effusion. Patients were monitored by telemetry until discharge the next day.

Oral anticoagulation was reinitiated at least 3 h after the procedure. It was continued for at least 2 months and thereafter according to the CHA2DS2-VA Score. Proton-pump inhibitors were prescribed in all patients for 6 weeks. AAD administration was discontinued immediately after ablation. In patients who underwent PVI combined with CTI ablation, kidney function markers such as serum creatinine, GFR (glomerular filtration rate), and cardiac enzymes [creatinine kinase (CK), creatinine kinase MB fraction (CK-MB)] were measured pre-ablation and at 24 h post-ablation to investigate acute kidney injury and cardiac enzyme kinetics of PFA for PVI and CTI ablation.

### 2.5. Endpoints

The primary objective of this study was to evaluate acute procedural success, defined by the complete isolation of all PVs, confirmed by both entry and exit blocks, along with the bidirectional linear anatomical block of any supplementary ablation lesions. The primary safety endpoint focused on identifying any predefined major complications related to the system and procedure, both during the intervention and the hospital stay. Secondary endpoints included procedural metrics such as the total duration, fluoroscopy time, and dose area product.

### 2.6. Statistical Analysis

Descriptive statistical analysis was performed to demonstrate outcomes and safety parameters associated with the novel intervention. Categorial variables are presented as numbers and percentages. Numerous data are summarized as mean with standard deviations. Differences between continuous variables were assessed with a t-test. Statistical calculations were performed with the statistical analysis software R (Version 4.2.0, 2022).

## 3. Results

### 3.1. Baseline Characteristics

In total, 100 consecutive patients with paroxysmal and persistent AF were included. The baseline characteristics are summarized in Table 1. Fifty percent of the patients had persistent AF. The mean age was 64 ± 13 years. A minority of the patients were female (29%). Most patients were overweight (BMI 29.0 ± 4.0 kg/m^2^). The mean LA area and LVEF were 24.2 ± 6.8 cm^2^ and 56 ± 6%, respectively. The time since the first AF diagnosis was 3.0 ± 4.5 years.

### 3.2. Procedural Data

**Figure 3:**
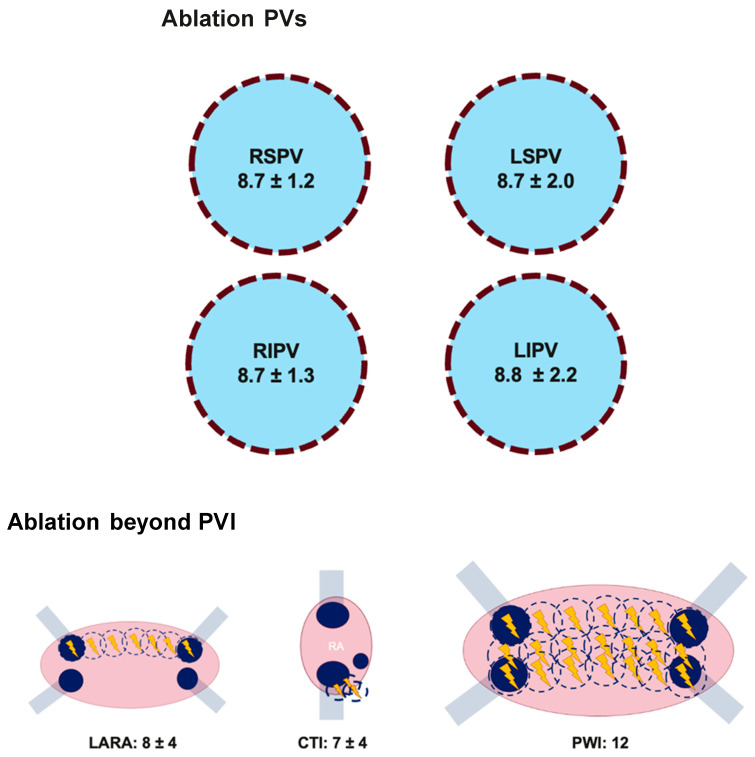
Applications for PVI and ablation beyond PVI. PV: pulmonary vein, PVI: pulmonary vein isolation, RSPV: right superior pulmonary vein, RIPV: right inferior pulmonary vein, LSPV: left superior pulmonary vein, LIPV: left inferior pulmonary vein, LARA: left atrial roof ablation, CTI: cavo-tricuspid ablation, PWI: posterior wall isolation.

Procedural data are shown in Table 2. Five patients underwent the procedure under general anesthesia. Out of 100 patients, 33 (33%) patients were treated with adjunctive LARA. Six (6%) additional CTI ablations using PFA were performed due to typical atrial flutter. In one patient, 3D anatomical mapping was performed before and after ablation. The skin-to-skin procedural time was 66.3 ± 13.8 min. The fluoroscopy time and dose area product were 13.7 ± 4.7 min and 6.8 ± 4.9 Gycm^2^, respectively. The total LA dwell time was 43.6 ± 10.5 min. Acute PVI was achieved in 100% of PVs. The number of PV applications was as follows: for the RSPV, the average was 8.7 ± 1.2 applications; for the RIPV, it was 8.7 ± 1.3 applications; the LSPV had an average of 8.7 ± 2.0 applications; and for LIPV, it was 8.8 ± 2.2 applications. The mean numbers of applications in left PVs and right PVs were 17.5 ± 3.6 and 17.2 ± 1.6 (*p* = 0.69), respectively. The total number of PV applications was 35.0 ± 3.1. LARA was successfully performed with 8.4 ± 3.6 applications (Figure 3). A bidirectional conduction block across the LA roof was confirmed in all 33 (100%) patients. Adjunctive PWI was performed in one persistent AF patient with 12 applications. Ablation of CTI was performed with 7.2 ± 3.8 applications, and afterwards, a bidirectional blockade was confirmed in all patients (100%). In 39 patients (39%), electrical cardioversion was required to restore the sinus rhythm. In one case, the initial attempt at electrical cardioversion was unsuccessful. Ultimately, the sinus rhythm was restored after the intravenous administration of 50 mg of flecainide and subsequent electrical cardioversion. During the one-day hospital stay, no arrhythmia recurrence was observed.

### 3.3. Complications

No major adverse events during procedure and hospital stay, including phrenic nerve injury, esophageal damage, or pericardial effusion, were reported in any of the patients. No conduction disturbances or ST elevations were observed during or after the ablation.

Two vascular access complications were observed as minor adverse events following the procedure (Table 3). One patient developed a hematoma, while another had an arteriovenous fistula. Both complications were reversible with compression using a pressure bandage, and no further intervention was required. Patients undergoing PVI and adjunctive CTI received 43 ± 3 PFA applications, and their renal function parameters were similar before and 24 h post-ablation (Table 4). As expected, cardiac enzyme levels significantly increased after ablation (*p* < 0.001).

## 4. Discussion

### 4.1. Main Findings

This is the first real-world report on PV ablation and non-PV-site ablation in patients with atrial fibrillation and flutter, using a novel circular array PFA catheter. All procedures were successful, achieving 100% complete PVI. Additional ablations were achieved on the LA roof, posterior wall, and CTI, exclusively using the PFA catheter. No major adverse events occurred, indicating a potentially outstanding safety profile for both PVI and extended ablation beyond PV sites.

### 4.2. Effectiveness of PVI and Ablation Beyond PVs

The acute efficacy of the PulseSelect™ system was demonstrated by the achievement of 100% PVI in all treated veins. This result underscores the acute effectiveness of the PFA technique in isolating PVs, a crucial endpoint for successful AF ablation. In all patients, PVI was achieved with a mean of 35 applications. The number and variability of applications between the left and right PVs exhibited numerical differences, although these differences were not statistically significant (17.5 ± 3.6 vs. 17.2 ± 1.6; *p* = 0.69). The left PVs are often anatomically more complex, exhibiting greater variability in their anatomy, which can complicate identification and isolation during ablation procedures. Additionally, the left upper pulmonary vein frequently branches into smaller vessels that also need to be isolated, further increasing the complexity of the treatment [6,7,8]. This complexity may contribute to the observed differences in the number of applications. Moreover, the ablations were conducted by multiple operators, which may have contributed to the observed variance.

The rationale for additional LARA in patients with persistent AF and significant LA enlargement is rooted in the understanding of AF substrate complexity. Persistent AF, particularly in the presence of a dilated left atrium, is often associated with a more extensive and diffuse fibrotic substrate, which can contribute to the perpetuation of arrhythmias even after successful PVI [9]. Targeting additional sites beyond the PVs, such as the LA roof, can help in modifying the atrial substrate to improve the outcomes in this challenging patient population [10].

However, several additional ablation strategies using radiofrequency (RF) technology for persistent AF have not demonstrated clear advantages in multicenter randomized trials [11,12,13,14,15]. Notably, the ERASE AF Trial, along with some single-center randomized and non-randomized studies, found that ablation beyond PVI could reduce atrial arrhythmia recurrence [15,16,17]. Yet, it is important to note that all these studies employed RF tip catheters. In a few trials, additional substrate modification, such as roof line or posterior wall isolation using cryoballoon (CB) ablation, has shown benefits in reducing atrial arrhythmia recurrence [10,18,19,20,21]. Nevertheless, data on the efficacy and safety of PFA beyond PVI are still limited. Recent data demonstrate that ablation of non-PV sites such as the roof line, posterior wall, or superior veins is both feasible and safe for AF patients [22,23]. Additionally, CTI ablation using single-shot PFA devices has proven to be effective in patients [24]. However, data on ablation beyond PVI using this novel circular array PFA ablation system do not exist. For the first time, we report a 100% conduction block after LARA and CTI ablation, which demonstrated the feasibility and acute efficacy of this novel ablation system.

Compared to thermal ablation sources (RF or CB), all procedural times—including skin-to-skin time, LA dwell time, and fluoroscopy time—were shorter, even compared to patients undergoing CB pulmonary vein isolation (PVI) combined with LARA [18,25]. In contrast to the Pulsed AF pivotal trial, shorter procedure and fluoroscopy times were observed. However, it is important to note that 3D mapping and a 20 min waiting period were utilized, and the procedures were performed at different centers by various operators in that pivotal trial [5]. In comparison to other studies and PFA ablation systems, the procedure time, fluoroscopy time, and total area dose product were longer in our patient population [3,26]. This is likely due to initial experiences with a novel PFA system, the performance of ablation beyond PVI in a significant number of patients, and the exchange of a transseptal sheath for a steerable sheath. However, the use of various 3D mapping systems in some previous studies complicates direct comparisons [27].

### 4.3. Safety

Thermal modes of ablation (cryoablation, RF) have performed reasonably for years, but are limited by the potential for collateral tissue damage (e.g., esophagus and phrenic nerve). PFA is based on the process of electroporation. Selective myocardial specific ablation is feasible without collateral damage [28,29]. Hence, thermal specific complications, such as phrenic nerve palsy, PV stenosis, or esophageal injuries, are unlikely. In previous preclinical and clinical trials, the safety profile of PFA was confirmed, including this novel circular PFA system [4,5]. In this study, no significant adverse events were observed. Notably, there were no thermal complications. Although ablations were performed beyond PVI, there were no occurrences of ST elevation, coronary spasm, or conduction disturbances. To date, the high safety of PWI has been reported in clinical trials [30,31]. Previous studies investigating other PFA systems have reported cases of coronary spasms, especially when performing a mitral isthmus line or CTI line [32]. Interestingly, in our study with the circular PFA system, only vascular complications were observed, unlike in the MANIFEST-17K study with the pentaspline catheter, which reported rare but serious complications, including pericardial tamponade, stroke, and death. However, the significantly larger patient population in that multi-national survey makes direct comparison challenging [33].

Additionally, we observed stable renal function parameters both before and 24 h post-ablation in patients undergoing PVI combined with CTI, where an average of 43 PFA applications were performed. This suggests a potential absence of hemoglobinuria-induced acute kidney injury and underscores the safety profile of ablation beyond PVI using this PFA system. However, it should be noted that hemolysis parameters were not evaluated in this trial. To date, several trials have described hemolysis-associated acute kidney injury and its correlation with the number of PFA applications with a pentaspline PFA catheter [34,35].

### 4.4. Clinical Implications

This study presents initial real-world data from a single center after commercialization of the PulseSelect™ System. It also provides the first description of ablation beyond PVI using this innovative ablation technology. Future research is necessary focusing on evaluating the long-term safety and efficacy of this PFA system, specifically regarding freedom from AF and lesion durability of PVs and non-PV sites. To establish robust conclusions about the long-term effectiveness of PFA, additional randomized controlled trials are needed. These trials should compare the PulseSelect™ System with conventional thermal ablation technologies and other PFA devices to identify any differences in efficacy and safety between these ablation approaches. This includes investigating the inflammatory remodeling processes in the atria following applications and evaluating the duration of the blanking period across different PFA catheters and energy sources [36].

### 4.5. Study Limitations

This study has several limitations. This is an observational single-center study with a new ablation system. Furthermore, the patient cohort is small. Moreover, there is a lack of follow-up after the hospital stay. Mid- and long-term data are needed to evaluate lesion durability and atrial arrhythmia recurrence.

## 5. Conclusions

In this study, the novel circular array PFA catheter demonstrated high feasibility, safety, and acute efficacy for PVI, additional LARA, and CTI ablation in a real-world setting. The system can be seamlessly integrated into standard PVI workflows, providing a promising alternative to traditional thermal ablation methods. Further large-scale studies are warranted to evaluate the long-term clinical outcomes and safety of this novel PFA system.

## Figures and Tables

**Table 1:** Baseline characteristics.

Patient Characteristics	*N* = 100
Female gender, *n*	29 (29%)
Age, years	64 ± 13
Persistent AF, *n*	50 (50%)
BMI, kg/m^2^	29.0 ± 4.0
CHA2DS2VA-Score	2.1 ± 1.5
Duration since first AF diagnosis, years	3.0 ± 4.5
Left atrial area, cm^2^	24.2 ± 6.8
Left ventricular ejection fraction, %	56 ± 6
Congestive heart failure, *n*	13 (13%)
Hypertension, *n*	69 (69%)
Diabetes mellitus, *n*	13 (13%)
Obstructive sleep apnea, *n*	8 (8%)
Coronary artery disease, *n*	31 (31%)
Pacemaker/Implantable defibrillator, *n*	2 (2%)
Creatinine, mg/dL	0.99 ± 0.2
GFR, mL/min/1.73 m^2^	79.09 ± 22.6
Hemoglobin, mg/dL	14.6 ± 1.3
History of stroke/TIA, *n*	9 (9%)
Previous anti-arrhythmic drugs	
Class I, *n*	8 (8%)
Class II, *n*	76 (76%)
Class III, *n*	18 (18%)
Class IV, *n*	1 (1%)

Data are expressed as mean ± standard deviation, or *n* (percentage). BMI: body mass index; GFR: glomerular filtration rate; TIA: transient ischemic attack.

**Table 2:** Procedural data.

Procedural Characteristics	*N* = 100
General anesthesia (intubation), *n*	5 (5%)
Deep sedation (no intubation), *n*	95 (95%)
Initial rhythm	
● Sinus rhythm, *n*	54 (54%)
● Atrial fibrillation, *n*	45 (45%)
● Atrial flutter, *n*	1 (1%)
Transseptal puncture, *n*	1 ± 0
3D electroanatomical mapping, *n*	1 (1%)
Procedural time (skin-to-skin), min	66.3 ± 13.8
Total LA-dwell time, min	43.6 ± 10.5
Total fluoroscopy time, min	13.4 ± 4.7
Total area dose, Gycm^2^	6.8 ± 4.9
Contrast medium, mL	21.0 ± 8.7
Intraprocedural cardioversion	39 (39%)

Data are expressed as mean ± standard deviation, or n (percentage). PVs: pulmonary veins, LARA: left atrial roof ablation, TIA: transient ischemic attack, PNP: phrenic nerve palsy.

**Table 3:** Adverse events. Data are expressed as *n* (percentage).

Adverse Events	*N* = 100
Major adverse events	
Pericardial tamponade, *n*	0
Coronary artery spasm, *n*	0
Cerebrovascular accident, *n*	0
Sustained phrenic nerve palsy, *n*	0
Atroesophageal fistula, *n*	0
Thermal esophageal injury, *n*	0
Pulmonary vein stenosis, *n*	0
Major bleeding requiring transfusion, *n*	0
Vascular access complications requiring intervention, *n*	0
Death, *n*	0
Minor adverse events	
Pericardial effusion (no requiring intervention), *n*	0
Transient ischemic attack, *n*	0
Transient phrenic nerve palsy, *n*	0
Vascular access complications (no requiring intervention), *n*	2 (2%)
Transient ST elevation, *n*	0

**Table 4:** Labor parameters in patients undergoing pulmonary vein isolation and cavo-tricuspid isthmus ablation. Data are expressed as mean ± standard deviation. GFR: glomerular filtration rate, CK: creatine kinase.

Variables	Baseline (*N* = 6)	24 h (*N* = 6)	*p*-Value
Creatinine, mg/dL	1.08 ± 0.11	1.04 ± 0.09	0.563
GFR, mL/min/1.73 m^2^	76.55 ± 11.21	77.51 ± 8.74	0.898
CK, U/L	150 ± 42	463 ± 79	<0.001
CK-MB, U/L	18 ± 3	53 ± 8	<0.001

## Data Availability

The data presented in this study are available on request from the corresponding authors.
